# DJ-1 exerts anti-inflammatory effects and regulates NLRX1-TRAF6 via SHP-1 in stroke

**DOI:** 10.1186/s12974-020-01764-x

**Published:** 2020-03-09

**Authors:** Li Peng, Yang Zhou, Ning Jiang, Tingting Wang, Jin Zhu, Yanlin Chen, Linyu Li, Jinyan Zhang, Shanshan Yu, Yong Zhao

**Affiliations:** 1 Department of Pathology, Chongqing Medical University, Yixueyuan Road 1, Chongqing, 400016 People’s Republic of China; 2 Molecular Medical Laboratory, Chongqing Medical University, Chongqing, 400016 People’s Republic of China; 3 Institute of Neuroscience, Chongqing Medical University, Chongqing, 400016 People’s Republic of China; 4 Key Laboratory of Neurobiology, Chongqing Medical University, Chongqing, 400016 People’s Republic of China

**Keywords:** Astrocyte, DJ-1, NLRX1-TRAF6, SHP-1, Inflammation, I/R injury

## Abstract

**Background:**

Acute inflammation induced by reactive astrocytes after cerebral ischemia/reperfusion (I/R) injury is important for protecting the resultant lesion. Our previous study demonstrated that DJ-1 is abundantly expressed in reactive astrocytes after cerebral I/R injury. Here, we show that DJ-1 negatively regulates the inflammatory response by facilitating the interaction between SHP-1 and TRAF6, thereby inducing the dissociation of NLRX1 from TRAF6.

**Methods:**

We used oxygen-glucose deprivation/reoxygenation (OGD/R) in vitro in primary astrocyte cultures and transient middle cerebral artery occlusion/reperfusion (MCAO/R) in vivo to mimic I/R insult.

**Results:**

The inhibition of DJ-1 expression increased the expression of the inflammatory cytokines TNF-α, IL-1β, and IL-6. DJ-1 knockdown facilitated the interaction between NLRX1 and TRAF6. However, the loss of DJ-1 attenuated the interaction between SHP-1 and TRAF6. In subsequent experiments, a SHP-1 inhibitor altered the interaction between SHP-1 and TRAF6 and facilitated the interaction between NLRX1 and TRAF6 in DJ-1-overexpressing astrocytes.

**Conclusion:**

These findings suggest that DJ-1 exerts an SHP-1-dependent anti-inflammatory effect and induces the dissociation of NLRX1 from TRAF6 during cerebral I/R injury. Thus, DJ-1 may be an efficacious therapeutic target for the treatment of I/R injury.

## Introduction

Pathophysiological cascades involving inflammation are triggered by cerebral ischemia/reperfusion (I/R)-induced neuronal death [1, 2]. Cerebral ischemia induces an inflammatory response characterized by the activation of astrocytes and microglia and the elevated production and release of inflammatory cytokines and chemokines that aggravate tissue damage [3, 4]. Although microglia are the first cells to respond to inflammation induced by cerebral ischemia, the responses of astrocytes to proinflammatory cytokines may also be relevant to tissue damage [5, 6]. The response of astrocytes to inflammation involves the loss of vital functions or the acquisition of injurious functions that aggravate inflammation and delay ischemic recovery [7]. Acute inflammation elicited by reactive astrocytes after insult is an important response for protecting and repairing the lesion [8]. In addition, utilizing the secretome of reactive astrocytes has been determined to be a therapeutic approach for reducing inflammation [9]. Knowledge of the mechanism of the astrocytic inflammatory response may allow the development of an efficacious therapeutic strategy to alleviate brain injury in stroke.

NLRX1 is a recently characterized member of the NOD-like family that is widely expressed in mitochondria in all tissues [10]. NLRX1, as an anti-inflammatory regulator, attenuates antimicrobial immune responses [11–13] and sterile inflammation [14] by inhibiting the NF-κB and RIG-1-MAV signaling pathways. NLRX1 was also recently shown to exert negative effects on inflammatory responses in the central nervous system (CNS) [15]. NLRX1−/− mice show upregulated NF-κB signaling, which contributes to neural tissue damage [16]. In unstimulated cells, NLRX1 associates with TRAF6. However, after cells are stimulated by lipopolysaccharide (LPS), NLRX1 rapidly dissociates from TRAF6 and inhibits TLR-mediated NF-κB activation and proinflammatory cytokine release [17]. Therefore, NLRX1, an anti-inflammatory agent that dissociates from TRAF6, is critical in regulating inflammation.

DJ-1 (also known as PARK7) has been linked to an early-onset autosomal recessive form of PD [18] and is abundantly expressed in reactive astrocytes in Parkinson’s disease [19]. Our previous study showed that upregulated astroglial DJ-1 in the infarct region plays a critical role in astrocyte neuroprotection after stroke [20], suggesting that DJ-1 affects the function of astrocytes. DJ-1 knockout (KO) astrocytes exhibit increased LPS-induced expression of proinflammatory mediators, which aggravate inflammatory damage induced by IFN-γ [21]. Tumor necrosis factor-α (TNF-α) has been found to be increased in injured DJ-1 KO brains [21]. DJ-1 regulates TRAF6 signals in bone marrow macrophages (BMMs) via Src homology region 2 domain-containing phosphatase-1 (SHP-1) [22]. In addition, astroglial DJ-1 exerts anti-inflammatory effects by promoting the interaction between SHP-1 and STAT1 [23]. However, to our knowledge, the detailed mechanisms by which DJ-1 plays this anti-inflammatory role in cerebral I/R injury remain unclear.

Previously, we reported that astroglial DJ-1 plays a critical role in neuroprotection in ischemic injury [20]. These findings led us to further study the protective mechanism of astroglial DJ-1. In this study, we demonstrate that DJ-1 plays anti-inflammatory roles in astrocytes. DJ-1 induces the dissociation of NLRX1 from TRAF6 by facilitating the interaction between SHP-1 and TRAF6. Thus, it is necessary to determine the anti-inflammatory function of astroglial DJ-1 in I/R insult.

## Methods

### Experimental animals and reagents

Adult male Sprague-Dawley (SD) rats (weighing 250–280 g) were obtained from the Animal Experimental Center of Chongqing Medical University and used for the in vivo study. Primary astrocytes were extracted from the cerebral cortices of newborn SD rats and cultured.

Glucose-free Dulbecco’s modified Eagle’s medium, Dulbecco’s modified Eagle’s medium (DMEM)/F12, and fetal bovine serum (FBS) were purchased from Gibco (Grand Island, NY, USA). Trypsin and Hank’s solution were obtained from HyClone (Logan, UT, USA). Penicillin/streptomycin (Pen/Strep) and phosphate-buffered saline solution (PBS) were obtained from Beyotime (Shanghai, China). Poly-L-lysine was purchased from Sigma-Aldrich (Milan, Italy). IL-1β, IL-6, and TNF-α enzyme-linked immunosorbent assay (ELISA) kits were obtained from Boster (Wuhan, China), and a CCK-8 kit was obtained from Dojindo (Kumamoto, Japan). TPI-1 was purchased from MedChem Express (NJ, USA).

### Middle cerebral artery occlusion/reperfusion (MCAO/R) model and TPI-1 dosing

Transient MCAO/R was performed as previously described by our laboratory [24]. Briefly, adult male SD rats were deprived of food and water for 8 h before operation, anesthetized with 3.5% chloral hydrate (350 mg/kg, intraperitoneal injection), and then placed on a heating pad to maintain their body temperature at 37 ± 0.5 °C for the operation. A nylon filament (Beijing Cinontech Co., Ltd., Beijing, China) was inserted into the left middle cerebral artery. Then, the filament was removed after 1 h of ischemia to allow reperfusion. Local cerebral blood flow (CBF) was monitored by a laser Doppler flowmeter (Periflux System 5000, Perimed, Sweden) during the operation. After reperfusion for 24 h, neurological deficit score assays were used to evaluate the success of the MCAO model, and brain tissues were subjected to Western blot analysis, immunoprecipitation, and immunohistochemistry. Sham-operated animals underwent the same surgery as the MCAO/R group, except they were not subjected to MCAO.

TPI-1 was obtained from MedChem Express. The SHP-1 inhibitor TPI-1 (HY-100463, MedChem Express) was dissolved in 10% DMSO and diluted to the final concentration in 90% saline (20% SBE-β-CD in saline). We intraperitoneally injected 1 mg/kg TPI-1 into MCAO rats after they awoke. An identical volume of DMSO was injected intraperitoneally as a control.

All analyses conducted in SD rats (*N* = 377) were divided into two sets of experiments; the first part was performed to study the effect of DJ-1 on inflammation after cerebral I/R, and the second part was performed to study the mechanism by which DJ-1 regulates the NLRX1-TRAF6 interaction. The first part comprised 4 groups, and the second part comprised 8 groups. The groups in the first part were as follows: (1) the sham group (*n* = 37, 1 died), (2) the MCAO group (*n* = 39, 3 died), (3) the NC group (MCAO + negative control siRNA) (*n* = 40, 4 died), and (4) the DJ-1 siRNA group (MCAO + DJ-1 siRNA) (*n* = 42, 6 died). The groups in the second part were as follows: (1) the sham group (*n* = 24, 0 died), (2) the MCAO group (*n* = 26, 2 died), (3) the scramble group (MCAO + scramble) (*n* = 28, 4 died), (4) the overexpression group (MCAO + DJ-1 overexpression) (*n* = 28, 4 died), (5) the DMSO group (MCAO + DMSO) (*n* = 27, 3 died), (6) the TPI-1 group (MCAO +TPI-1) (*n* = 27, 3 died), (7) the overexpression + DMSO group (MCAO + DMSO + DJ-1 overexpression) (*n* = 30, 6 died), and (8) the overexpression + TPI-1 group (MCAO +TPI-1 + DJ-1 overexpression) (*n* = 29, 5 died). In our study, 6 rats were used per experimental group, and each technique was performed in at least triplicate.

### Treatment with DJ-1 siRNA or DJ-1 adeno-associated virus (AAV)

DJ-1 siRNA was obtained and used as described in our previous studies [20]. AAV was purchased from Neuron Biotech (Shanghai, China). The CDS sequences (NM_001277249) were used to overexpress the DJ-1 gene in rat. The scramble AAV did not contain inserts. One month before MCAO, AAV was injected into the left lateral cerebral cortices of the rats. The overexpression efficiency of DJ-1 AAV was confirmed by Western blot analysis.

### Evaluation of neurological deficits

To assess neurological deficits in the rats on day 1 after MCAO/R, a modified scoring system was applied as described in our previous studies [20]. The scoring system was as follows: grade 0, no neurological damage; grade 1, failure to extend the contralateral forelimb fully; grade 2, circling to right; grade 3, falling to the right; grade 4, no spontaneous autonomic activity or loss of consciousness; grade 5, death.

### Quantification of infarct volume

After MCAO/R, brains were sliced into 2-mm-thick sections and stained with 2% 2,3,5-triphenyltetrazolium chloride (TTC, Sigma, USA) at 37 °C for 30 min as previously described [20, 25]. Then, the sections were fixed in 4% paraformaldehyde at 4 °C for 24 h. Each section was photographed, and the volume was quantified using ImageJ (version 6.0, NIH, Bethesda, MD, USA). The infarct volume was calculated using the following equation: {[total infarct volume − (ipsilateral hemisphere volume contralateral hemisphere volume)]/contralateral hemisphere volume × 100%.

### HE and Nissl staining

After neurological examination following MCAO/R, the rats were sacrificed with an overdose of 3.5% chloral hydrate, and their brains were perfused transcardially with 4% paraformaldehyde. The brains were dehydrated and embedded in paraffin after immersion in paraformaldehyde for 24 h. Then, 5-μm coronal sections were stained with hematoxylin and eosin (HE) or 0.1% cresyl violet (Nissl staining) based on standard protocols and mounted for microscopy.

### Primary rat cortical astrocyte culture and oxygen and glucose deprivation/reoxygenation (OGD/R) treatment

Astrocytes were cultured as previously described [20]. Briefly, cortical astrocytes were cultured in 75 cm^2^ flasks at a density of 2.0 × 10^7^/well in DMEM/F12 medium with 10% fetal bovine serum and 1% pen/strep in a humidified incubator in 5% CO_2_ at 37 °C. After the cells were cultured for 2–3 weeks, the microglia were removed from the flasks by mild shaking, the remaining adherent astrocytes were detached with trypsin, and the recovered cells were plated directly on the bottom of 6-well plates. Astrocytes that spread over the bottom of the plates (7–9 days) were used for further study. Astrocyte purity was determined using the astrocyte-specific marker GFAP.

OGD/R was conducted as previously described. Briefly, astrocytes were established in glucose-free DMEM and were transferred to an incubator with 94% N_2_, 1% O_2_, and 5% CO_2_ at 37 °C for 5 h. The glucose-free DMEM was changed to normal culture medium (DMEM/F12 medium containing 10% fetal bovine serum and 1% pen/strep), and then TPI-1 (10 ng/ml) was added to the culture medium in the 6-well plates. TPI-1 was dissolved in 10% DMSO and diluted to the final concentration in PBS. An identical volume of DMSO and PBS was added to the culture medium as a control. Next, the cells were grown in an incubator with 5% CO_2_ for 24 h at 37 °C. All the analyses conducted in astrocytes were divided into two sets of experiments; the first part was performed to study the effect of DJ-1 on inflammation after cerebral I/R, and the second part was performed to study the mechanism by which DJ-1 regulates the NLRX1-TRAF6 interaction. The first part comprised 4 groups, and the second part comprised 8 groups. The following groups were used in the first part: (1) the control group, (2) the OGD/R group, (3) the NC group (OGD/R + negative control siRNA), and (4) the DJ-1 siRNA group (OGD/R + DJ-1 siRNA). The following groups were used in the second part: (1) the control group, (2) the OGD/R group, (3) the scramble group (OGD/R + scramble), (4) the overexpression group (OGD/R + DJ-1 overexpression), (5) the DMSO group (OGD/R + DMSO), (6) the TPI-1 group (OGD/R + TPI-1), (7) the overexpression + DMSO group (OGD/R + DMSO + DJ-1 overexpression), and (8) the overexpression + TPI-1 group (OGD/R + TPI-1 + DJ-1 overexpression). Astrocytes were cultivated with 100 nM TPI-1 after OGD. In our study, 6 cultures were performed per experimental group, and each technique was performed in at least triplicate.

### DJ-1 interference and overexpression in astrocytes

A lentivirus was obtained and used as described in our previous studies [20]. The following sequence was designed and synthesized to knock down DJ-1: 5-CCGGACGGCAGTCACTACAGCTACTCAAGAGA TAGCTGTAGTGACTGCCGTTTTTTTG-3. The control sequence for DJ-1 knockdown was 5-CCGGTTCTCCGAACGTGTCACGTTTCAAGAGA ACGTGACACGTTCGGAGAATTTTTTG-3. The sequence used to overexpress DJ-1 was 5-ATGGACTACAAGGATGACGATGACAAGGATTACAAAGACGACGATGATAAGGACTATAAGGATGATGACGACAAA-3. The scramble lentivirus did not contain inserts. Three days before OGD/R, lentivirus was added to the culture medium. OGD/R was performed after astrocyte transfection. Western blotting was used to assess the transfection efficiency of the lentivirus.

### Cell viability

To assess astrocyte viability, we used a CCK-8 assay kit (Dojindo, Kumamoto, Japan). Astrocytes were cultured in 96-well plates. Three days before OGD/R, the astrocytes were treated with lentivirus for 72 h. Then, the cells were subjected to OGD/R, and the CCK-8 assay was performed. CCK-8 solution was added to the medium (10 μl/mL). After the astrocytes were cultured with medium at 37 °C for up to 2 h, they were measured with a microplate reader (Bio-Rad, Foster City, CA, USA) at an absorbance of 450 nm.

### Immunocytochemistry and immunohistochemistry

Frozen sections and cells on coverslips were fixed in 4% paraformaldehyde and then washed with PBS. After blocking with 5% FBS/0.01% Triton X-100 in PBS at 37 °C for 1 h, the cells were incubated with the following primary antibodies overnight at 4 °C: NLRX1 (1:100, Abcam, ab105412) and Hsp60 (1:100, Proteintech, 66041-1-1 g). The next day, the cells and sections were incubated in a DyLight 488-conjugated goat anti-mouse IgG antibody (1:200, Abbkine, green, A23210) and a DyLight 549-conjugated goat anti-rabbit IgG antibody (1:200, Abbkine, red, A23320) at 37 °C for 30 min. Vectashield containing DAPI was used to mount the coverslips and sections. A laser scanning confocal microscope (LSCM) was used to visualize the sections and glass slides.

### Western blot analysis

Total protein from cultured astrocytes and ischemic brain tissues was prepared using RIPA buffer with the protease inhibitor PMSF. A total of 50 μg of protein (per lane) was separated by SDS-PAGE (different percentages of SDS-PAGE gels were used to separate and analyze the different proteins) and electrotransferred to polyvinylidene fluoride (PVDF) membranes (Millipore, Boston, MA, USA). The membranes were then blocked with 5% nonfat milk in TBST buffer for 2.0 h at 37 °C and incubated with the following primary antibodies overnight at 4 °C: DJ-1 (1:1000, Abcam, ab76008), anti-β-actin (1:3000, Proteintech, 66009-1-Ig), IL-1β (1:200, Boster, PB0055), IL-6 (1:200, Boster, PB0061), TNF-α (1:200, Boster, PB0082), NLRX1 (1:200, Finetest, FNab05759), TRAF6 (1:200, Santa Cruz Biotechnology, sc-8409), and SHP-1 (1:200, Abcam, ab227503). The next day, the membranes were incubated with secondary antibodies at 37 °C for 2 h. The densities of the bands were detected using an imaging densitometer (Bio-Rad), and the gray values of the bands were quantified using ImageJ. Relative protein expression was normalized to β-actin staining intensity.

### Immunoprecipitation and immunoblot analyses

In the immunoprecipitation experiments, whole tissue and cell lysates were prepared after I/R and incubated with the indicated antibodies together with protein A/G beads (MedChem Express) overnight. NLRX1 (1:200, Finetest, FNab05759), TRAF6 (1:200, Santa Cruz Biotechnology, sc-8409), and SHP-1 (1:200, Abcam, ab227503) antibodies were used to immunoprecipitate NLRX1, TRAF6, and SHP-1, respectively. The beads were then washed 4 times with lysis buffer, and the immunoprecipitates were separated by SDS-PAGE. The proteins were transferred to PVDF membranes (Millipore, Boston, MA, USA) and then incubated with the indicated antibodies.

### ELISA measurements of TNF-α, IL-1β, and IL-6

After MCAO for 1 h and OGD for 5 h followed by reoxygenation for 24 h, total protein from the ischemic penumbra of the cerebral cortex and cultured astrocytes was prepared using RIPA buffer with the protease inhibitor PMSF. We used TNF-α, IL-1β, and IL-6 ELISA kits (Boster Institute, Wuhan, China) to measure the levels of TNF-α, IL-1β, and IL-6 expression in vivo and in vitro. We assessed the expression of these inflammatory cytokines in collected tissue and cell lysates according to the manufacturer’s instructions. The signal was measured at 450 nm.

### Statistical analysis

All data are shown as the means ± standard errors of the means, and statistical analysis was conducted using GraphPad Prism software (version 6.0). Statistical comparisons were assessed with one-way analysis of variance (ANOVA) followed by Tukey’s test. A value of *p* < 0.05 indicated statistical significance.

## Results

### DJ-1 interference aggravated injury after cerebral I/R injury

**Fig. 1:**
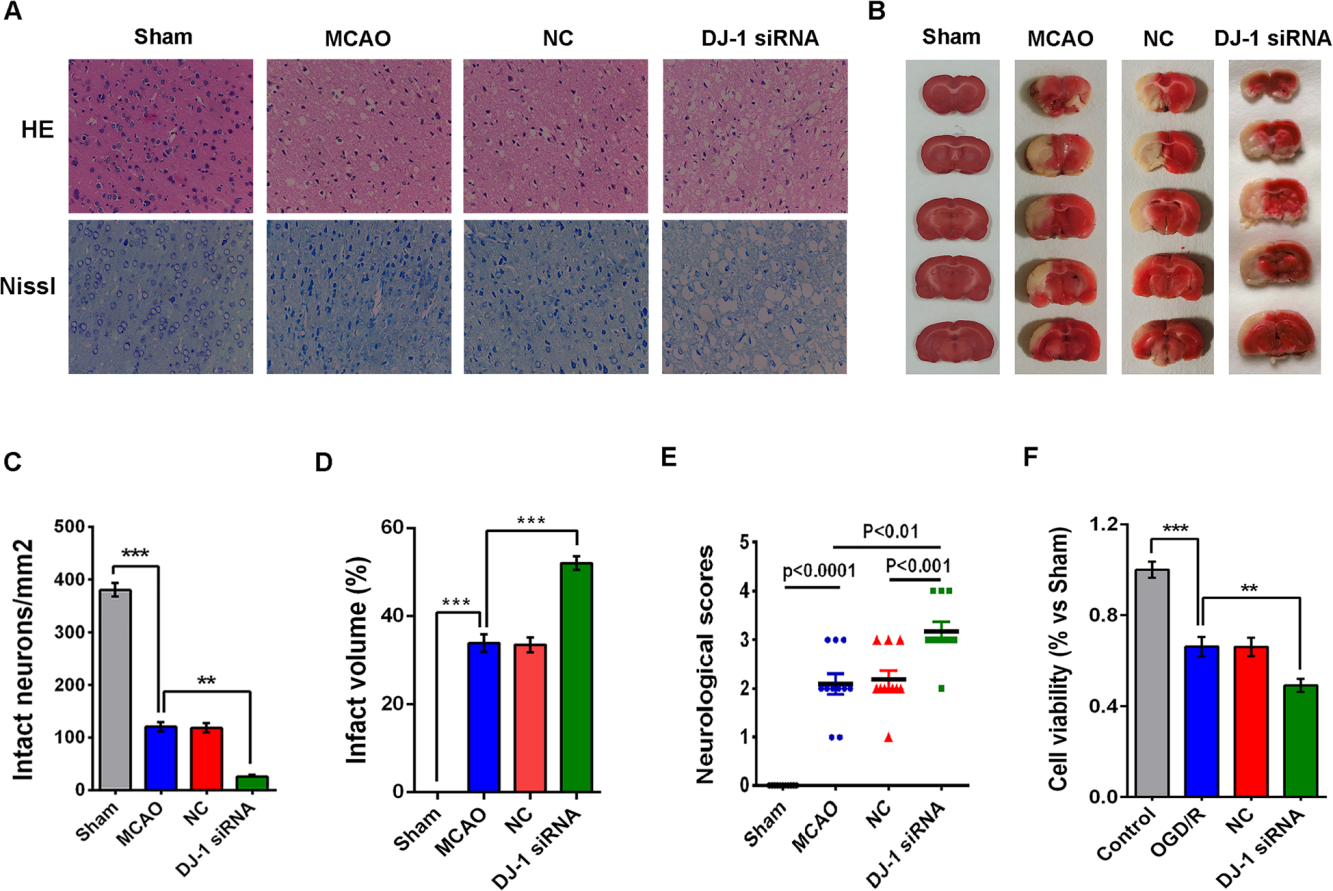
DJ-1 interference exacerbated injury after cerebral I/R injury. **a** HE staining and Nissl staining (× 400). **b** TTC staining. **c** Histograms showing the number of intact neurons in the cortex. **d** Infarct volume in the brain. **e** Neurological scores. **f** Cell viability after OGD/R. OGD/R = oxygen-glucose deprivation/reoxygenation. The in vivo groups were as follows: (1) the sham group, (2) the MCAO group, (3) the NC group, and (4) the DJ-1 siRNA group. The in vitro groups were as follows: (1) the control group, (2) the OGD/R group, (3) the NC group, and (4) the DJ-1 siRNA group. The data are expressed as the mean ± SEM. *p < 0.05, **p < 0.01, ***p < 0.001, ****p < 0.0001. *n* = 6 per group

HE and Nissl staining were conducted to assess morphological changes in the ischemic lesions 24 h after reperfusion (Fig. 1a, c). HE staining demonstrated that neurons in the MCAO and NC groups adopted a more disorderly arrangement than those in the sham group and exhibited loosened cytoplasm and karyopyknosis. However, transfection with DJ-1 siRNA increased the level of diffuse vacuolization and edema in the interstitial area and decreased the number of intact neurons. The results of Nissl staining were similar to those of HE staining. In contrast, a large number of atrophic neurons with damaged nuclei and shrunken cytoplasms were observed in the MCAO and NC groups. Compared to those in the MCAO group, most neurons in the DJ-1 interference group showed a loss of Nissl bodies. Cerebral infarct volumes and neurological deficit scores were used to further confirm the neuroprotective role of DJ-1. As shown in Fig. 1b, d, no infarction was observed in the sham group. The infarct volume was significantly increased in the DJ-1 siRNA group compared with the MCAO group. As shown in Fig. 1e, compared with those of the MCAO group, the neurological deficit scores of rats infected with DJ-1 siRNA were increased.

The CCK-8 assay was conducted to evaluate the roles of DJ-1 in astrocyte viability after exposure to OGD/R. Cells in the OGD/R group and NC group were less viable than those in the control group, and viability was further decreased in the DJ-1 siRNA group (Fig. 1f). These results suggest that DJ-1 interference can aggravate cerebral I/R injury.

### DJ-1 interference increased the expression of TNF-α, IL-1β, and IL-6 after cerebral I/R injury

**Fig. 2:**
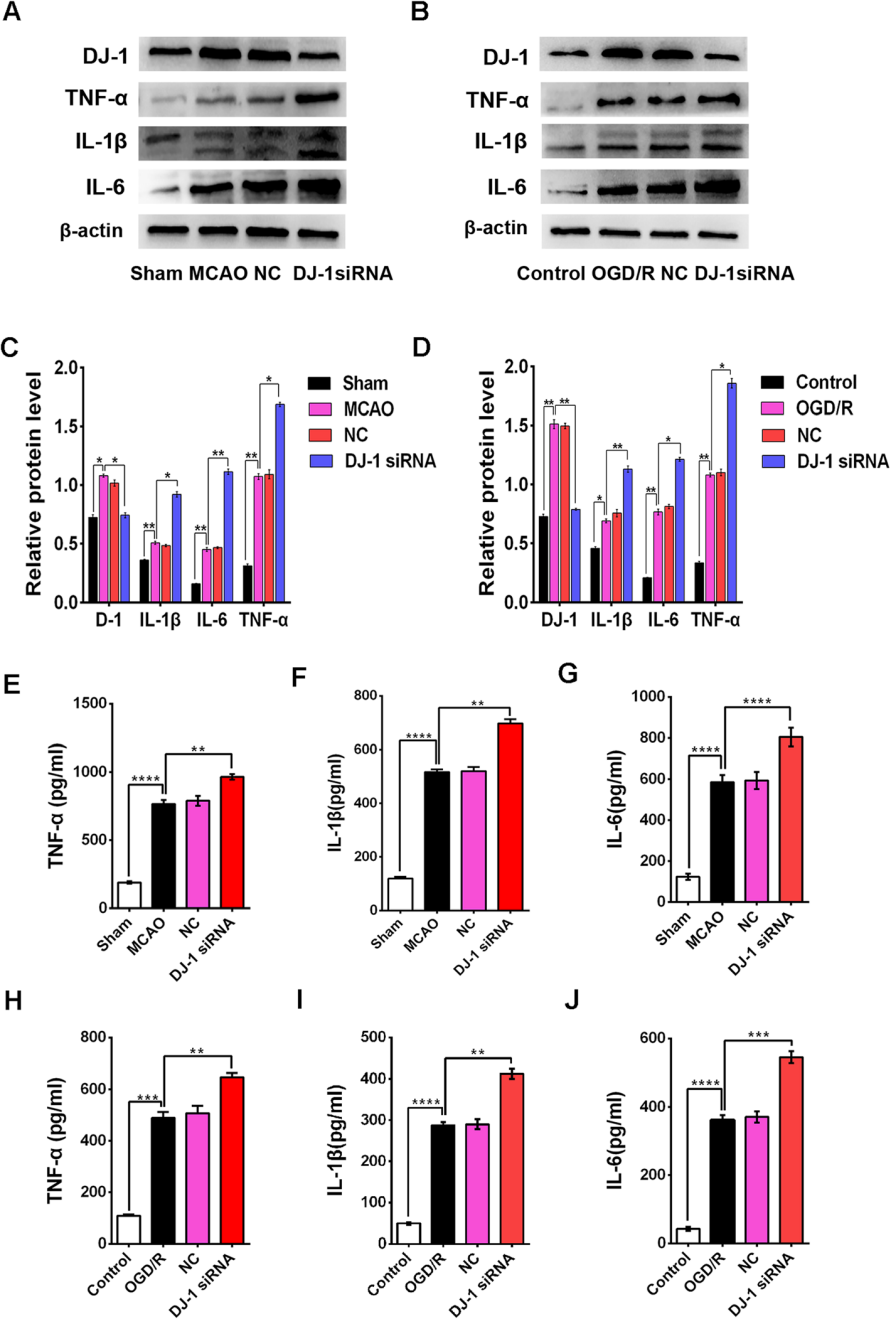
DJ-1 interference increased the expression of TNF-α, IL-1β, and IL-6 after cerebral I/R injury. **a** and **c** Western blot detecting DJ-1 and the cytokines TNF-α, IL-1β, and IL-6 in rats. **b**, **d** Western blot detecting DJ-1 and the cytokines TNF-α, IL-1β, and IL-6 in astrocytes. **e–g** Quantification of TNF-α, IL-1β, and IL-6 in rats by ELISA. **h‑j** Quantification of TNF-α, IL-1β, and IL-6 in astrocytes by ELISA. The data are expressed as the mean ± SEM. *p < 0.05, **p < 0.01, ***p < 0.001, ****p < 0.0001. *n* = 6 per group

To determine whether DJ-1 is involved in inflammation, we analyzed the expression of inflammatory cytokines after DJ-1 interference. Western blot analysis and ELISA were conducted to detect the expression levels of TNF-α, IL-1β, and IL-6. In vivo TNF-α, IL-1β, and IL-6 expression in the MCAO group was increased compared with that in the sham group, and the expression of these cytokines was further increased in the DJ-1 siRNA group (Fig. 2a, c, e‑g). Similar results were observed in vitro (Fig. 2b, d, h‑j).

### DJ-1 regulated NLRX1, TRAF6, and SHP-1 after cerebral I/R injury

**Fig. 3:**
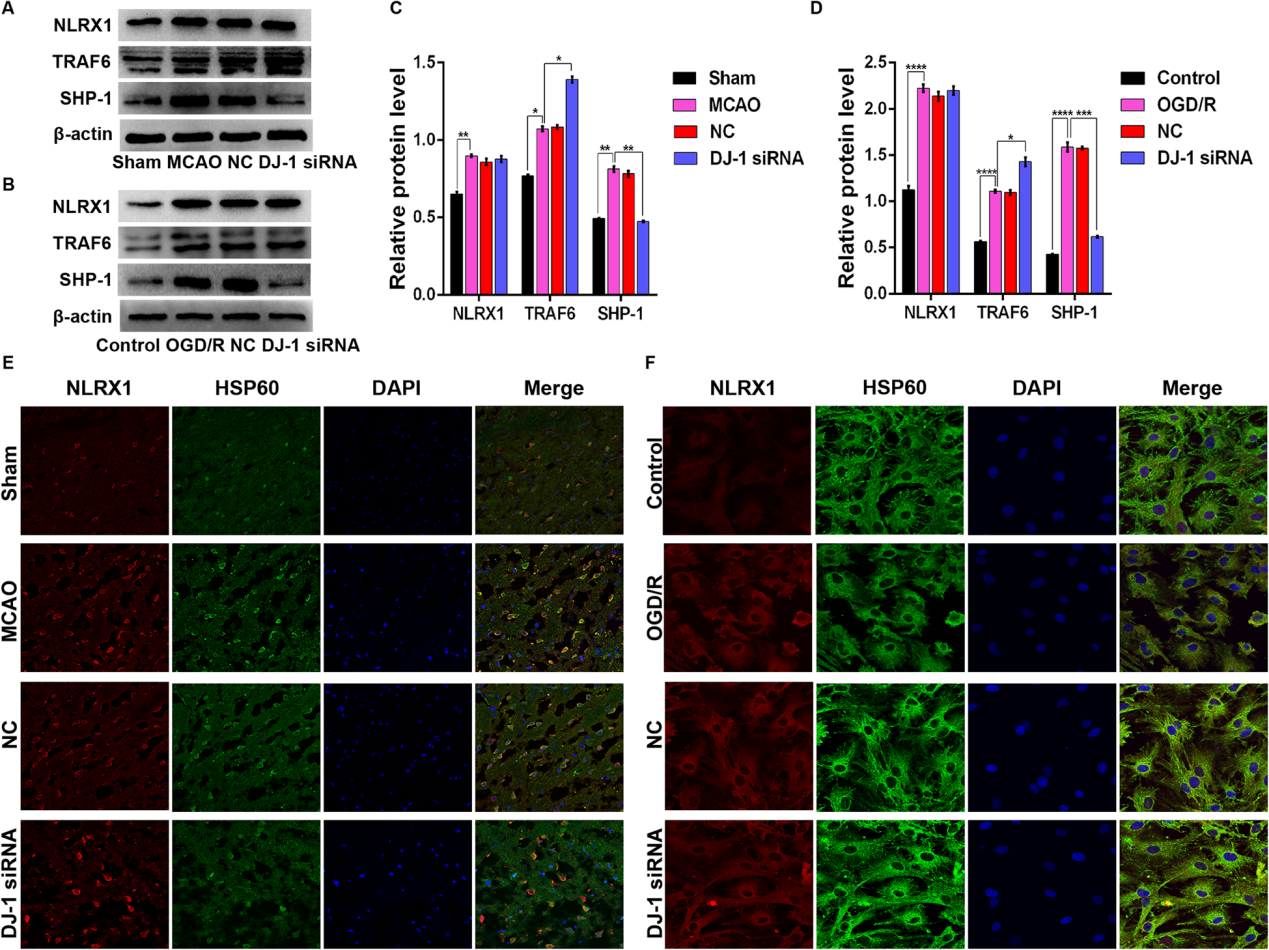
DJ-1 regulated the expression of NLRX1, TRAF6, and SHP-1 after cerebral I/R injury. **a**, **c** Western blot detecting NLRX1, TRAF6, and SHP-1 in rats. **b**, **d** Western blot detecting NLRX1, TRAF6, and SHP-1 in astrocytes. **e** Immunohistochemistry was used to measure NLRX1 expression in mitochondria in rats after DJ-1 knockdown. Original magnification, × 400. **f** Immunocytochemistry was used to measure NLRX1 expression in mitochondria in astrocytes. Original magnification, × 600. Fluorescence microscopy was used to assess NLRX1 expression, which is indicated by red fluorescence. HSP60 expression is indicated by green fluorescence. Cell nuclei were stained with DAPI. The data are expressed as the mean ± SEM. *p < 0.05, **p < 0.01, ***p < 0.001, ****p < 0.0001. *n* = 6 per group

NLRX1, a recently characterized member of the NOD-like family, is widely expressed in mitochondria. We assessed mitochondrial NLRX1 expression using the mitochondria-specific marker HSP60. As shown in Fig. 3e, f, NLRX1 was expressed in mitochondria. NLRX1, which is known to negatively regulate inflammation, also negatively regulates TLR-induced NF-κB signaling by dissociating from TRAF6 [17]. Because DJ-1 is also an anti-inflammatory regulator, we investigated whether DJ-1 regulates the expression of NLRX1 and TRAF6 after cerebral I/R injury. The expression of NLRX1 was significantly upregulated following cerebral I/R injury compared to the level in control samples. However, changes in DJ-1 had no effect on the levels of NLRX1 in vivo or in vitro (Fig. 3a‑d). After MCAO, the expression of TRAF6 was increased compared with that in the sham group and was further increased in the DJ-1 siRNA group (Fig. 3a, c). Similar results were observed in vitro (Fig. 3b, d). Similarly, SHP-1 acts as a critical negative regulator of the inflammatory response [26]; thus, we assessed the expression of SHP-1. After MCAO, the expression of SHP-1 was higher than that in the sham group, and after DJ-1 knockdown, the expression of SHP-1 was lower than that in the MCAO group (Fig. 3a, c). Similar results were observed in vitro (Fig. 3b, d).

### DJ-1 regulated the interaction between TRAF6 and NLRX1 and between TRAF6 and SHP-1 after cerebral I/R injury

**Fig. 4:**
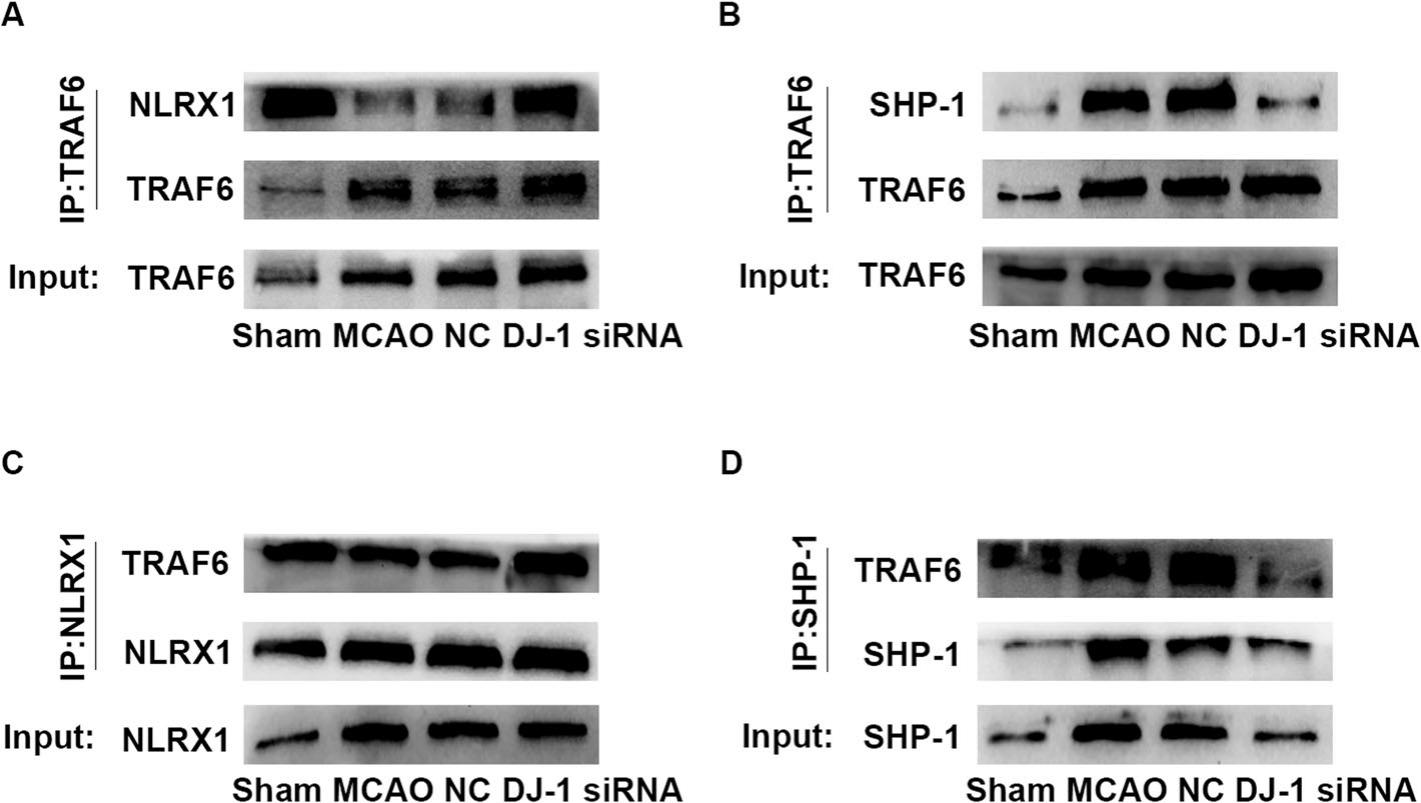
DJ-1 regulated SHP-1-TRAF6 and NLRX1-TRAF6 interactions after cerebral I/R injury. **a, c** Immunoprecipitation and immunoblot analyses of NLRX1-TRAF6 in rats. NLRX1 disassociated from TRAF6 after MCAO/R, and NLRX1 interacted with TRAF6 after treatment with DJ-1 siRNA. **b**, **d** Immunoprecipitation and immunoblot analyses of SHP-1-TRAF6 in rats. SHP-1 was associated with TRAF6 in the MCAO group, and SHP-1 disassociated from TRAF6 after treatment with DJ-1 siRNA. *n* = 6 per group

NLRX1 inhibits TLR-mediated NF-κB signaling by dissociating from TRAF6 [17]; thus, we examined whether DJ-1 regulates the interaction between NLRX1 and TRAF6 after cerebral I/R injury. NLRX1 interacted with TRAF6 in the sham group; however, NLRX1 disassociated from TRAF6 after cerebral I/R injury. Interestingly, DJ-1 interference promoted the interaction between NLRX1 and TRAF6 (Fig. 4a, c). These results indicate that NLRX1 may associate with TRAF6 in resting cells. After cerebral I/R injury, NLRX1 may disassociate from TRAF6 and thus inhibit inflammation. However, DJ-1 interference prevented the dissociation of NLRX from TRAF6 and thus enhanced inflammation. TRAF6 plays a prominent role in inflammation through NF-κB activation, and a previous study demonstrated an association between SHP-1 and TRAF6 in RANKL-stimulated BMMs [27]. Therefore, we hypothesized that TRAF6 interacts with SHP-1. We assessed the interaction between SHP-1 and TRAF6 after astroglial DJ-1 interference during cerebral I/R injury. SHP-1 was associated with TRAF6 in the MCAO group but not in the sham group. These interactions were significantly inhibited after treatment with DJ-1 siRNA (Fig. 4b, d).

As shown in Fig. 4a, c, DJ-1 interference promoted the interaction between NLRX1 and TRAF6. These results suggest that DJ-1 facilitates the dissociation of TRAF6 from NLRX1 and may be related to the interaction between TRAF6 and SHP-1.

### DJ-1 inhibited the expression of TNF-α, IL-1β, and IL-6 after cerebral I/R injury via SHP-1

**Fig. 5:**
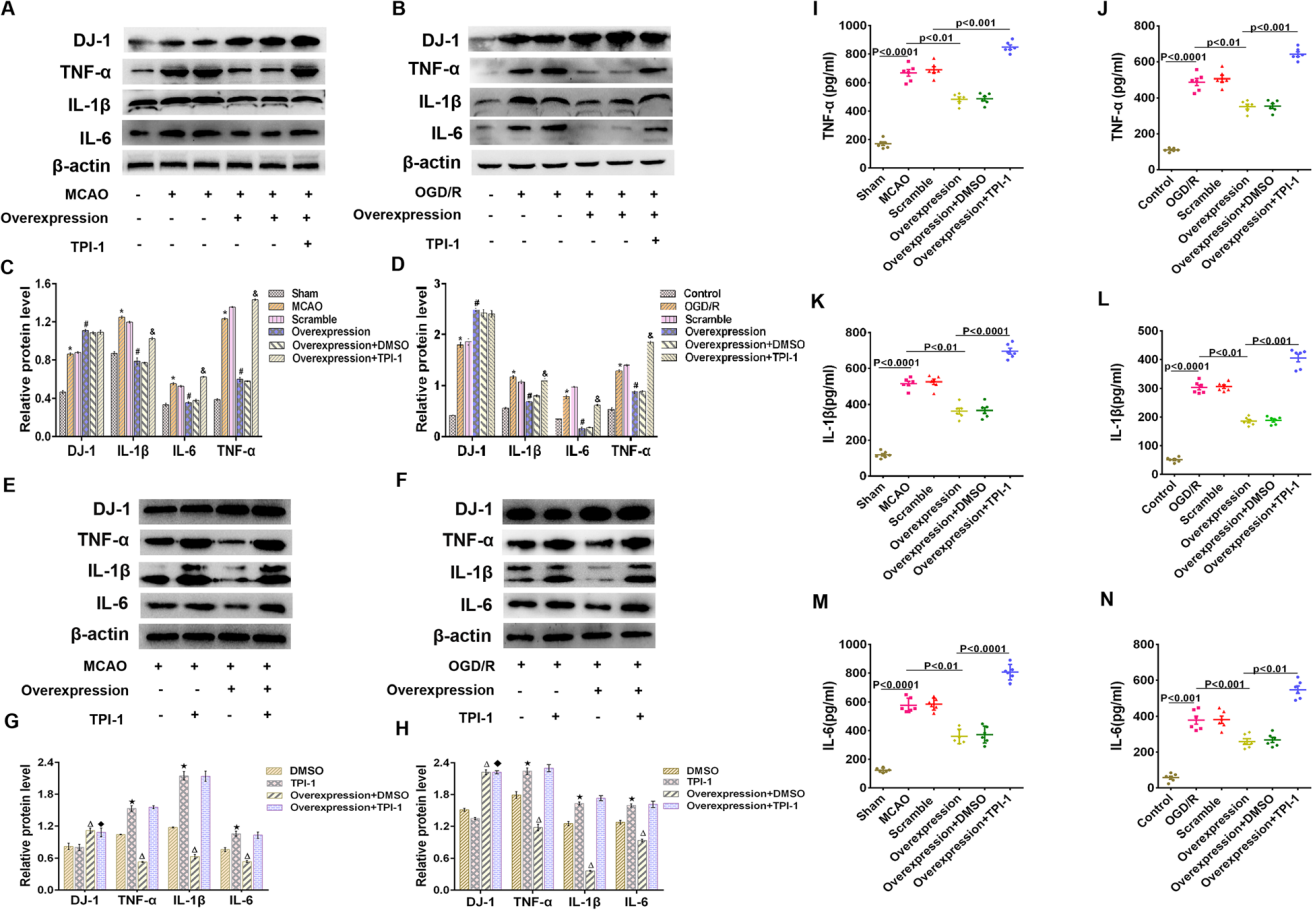
DJ-1 inhibited the expression of TNF-α, IL-1β, and IL-6 after cerebral I/R injury via SHP-1. **a**, **c** After virus and TPI-1 were used to overexpress DJ-1 and inhibit SHP-1, respectively, Western blotting was used to detect the cytokines IL-1β, IL-6, and TNF-α in rats. **b**, **d** After virus and TPI-1 were used to overexpress DJ-1 and inhibit SHP-1, respectively, Western blotting was used to detect the cytokines IL-1β, IL-6, and TNF-α in astrocytes. **e**, **g** After treatment with an SHP-1 inhibitor, Western blotting was used to detect the cytokines IL-1β, IL-6, and TNF-α in rats. **f**, **h** After treatment with an SHP-1 inhibitor, Western blotting was used to detect the cytokines IL-1β, IL-6, and TNF-α in astrocytes. **i**, **k**, **m** Quantification of TNF-α, IL-1β, and IL-6 in rats by ELISA. **j**, **l**, **n** Quantification of TNF-α, IL-1β, and IL-6 in astrocytes by ELISA. The data are expressed as the mean ± SEM. *p < 0.05 vs. the sham group; ^#^*p* < 0.05 vs. the MCAO group; ^&^*p* < 0.05 vs. the overexpression group; ^**★**^*p* < 0.05 vs. the DMSO group; ^**△**^*p* < 0.05 vs. the DMSO group; ^**◆**^*p* < 0.05 vs. the TPI-1 group. *n* = 6 per group. The data are expressed as the mean ± SEM. *p < 0.05 vs. the control group; ^#^*p* < 0.05 vs. the OGD/R group; ^&^*p* < 0.05 vs. the overexpression group; ^**★**^*p* < 0.05 vs. the DMSO group; ^**△**^*p* < 0.05 vs. the DMSO group; ^**◆**^*p* < 0.05 vs. the TPI-1 group. *n* = 6 per group. The groups in **a** and **b** and their corresponding groups in the statistical graphs are as follows: sham (−−−), MCAO or OGD/R (+−−), scramble (+−−), overexpression (++−), overexpression + DMSO (++−), and overexpression + TPI-1 (+++). The groups in **e** and **f** and the corresponding groups in the statistical graphs are as follows: DMSO (+−−), TPI-1 (+−+), overexpression + DMSO (++−), overexpression + TPI-1 (+++)

**Fig. 6:**
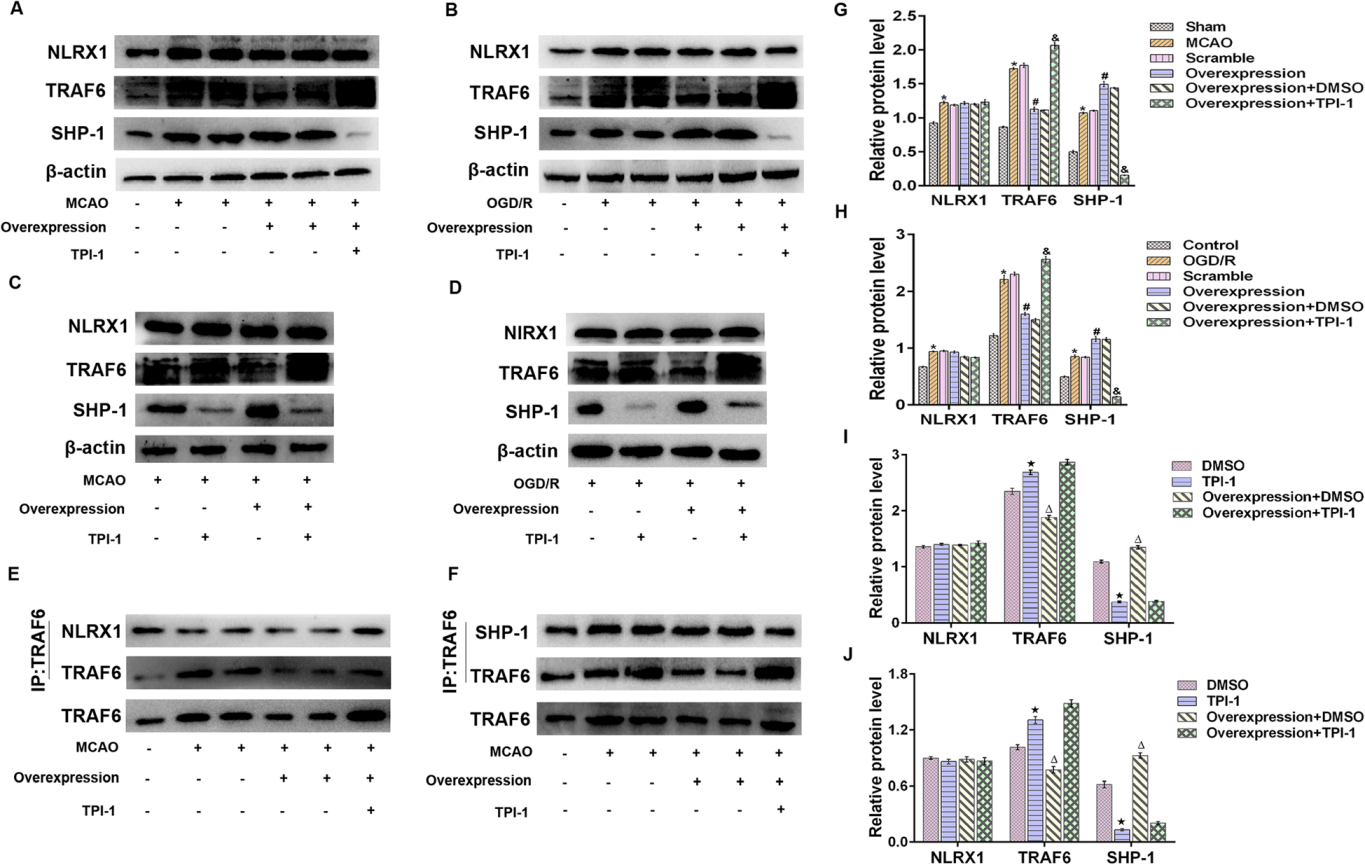
DJ-1 regulated the disassociation of NLRX1 from TRAF6 after cerebral I/R injury via SHP-1. **a**, **g** After virus and TPI-1 were used to overexpress DJ-1 and inhibit SHP-1, respectively, Western blotting was used to detect NLRX1, TRAF6, and SHP-1 in rats. **b**, **h** After virus and TPI-1 were used to overexpress DJ-1 and inhibit SHP-1, respectively, Western blotting was used to detect NLRX1, TRAF6, and SHP-1 in astrocytes. **c**, **i** After treatment with an SHP-1 inhibitor, Western blotting was used to detect the cytokines IL-1β, IL-6, and TNF-α in rats. **d**, **j** After treatment with an SHP-1 inhibitor, Western blotting was used to detect the cytokines IL-1β, IL-6, and TNF-α in astrocytes. **e** Immunoprecipitation and immunoblot analyses of NLRX1-TRAF6 in rats. **f** Immunoprecipitation and immunoblot analyses of SHP-1-TRAF6 in rats. The data are expressed as the mean ± SEM. *p < 0.05 vs. the sham group; ^#^*p* < 0.05 vs. the MCAO group; ^&^*p* < 0.05 vs. the overexpression group; ^**★**^*p* < 0.05 vs. the DMSO group; ^**△**^*p* < 0.05 vs. the DMSO group. *n* = 6 per group. The data are expressed as the mean ± SEM. *p < 0.05 vs. the control group; ^#^*p* < 0.05 vs. the OGD/R group; ^&^*p* < 0.05 vs. the overexpression group; ^**★**^*p* < 0.05 vs. the DMSO group; ^**△**^*p* < 0.05 vs. the DMSO group. *n* = 6 per group

SHP-1 is a strong negative regulator of inflammatory effects, and SHP-1-deficient mouse brains show enhanced inflammation [26]. To determine whether SHP-1 is involved in inflammation, TPI-1 was used to inhibit the expression of SHP-1. As shown in Fig. 6e‑h, inhibition of SHP-1 increased the expression of TNF-α, IL-1β, and IL-6 compared with that in the DMSO group in vivo and vitro. However, the inhibition of SHP-1 had no effect on the level of DJ-1. Interestingly, DJ-1 can regulate SHP-1, and we assessed whether DJ-1 inhibits cytokine levels after stroke via SHP-1. Virus and TPI-1 were used to overexpress DJ-1 and inhibit SHP-1. The overexpression of DJ-1 reduced the expression of TNF-α, IL-1β, and IL-6 compared with that in the MCAO group (Fig. 5a, c, i, k, m). After treatment with an SHP-1 inhibitor, the expression levels of TNF-α, IL-1β, and IL-6 were increased. Similar results were obtained in vitro (Fig. 5b, d, j, l, n). Thus, DJ-1 exerts anti-inflammatory effects in astrocytes during cerebral I/R injury via SHP-1.

### DJ-1 regulated the disassociation of NLRX1 from TRAF6 after cerebral I/R injury via SHP-1

To determine whether the DJ-1-induced dissociation of NLRX1 from TRAF6 occurs via SHP-1, Western blotting was conducted to assess the expression of NLRX1, TRAF6, and SHP-1, and immunoprecipitation was used to detect the interactions between NLRX1 and TRAF6 and between TRAF6 and SHP-1. As shown in Fig. 6a–d, g–j, the overexpression of DJ-1 increased SHP-1 levels, and treatment with TPI-1 reduced SHP-1 levels in vivo and vitro. However, changes in DJ-1 or SHP-1 levels had no effect on the levels of NLRX1 in vivo or in vitro. Figure 6a, g shows that overexpressing DJ-1 reduced TRAF6 expression compared with that in the MCAO group, and TRAF6 expression was increased after treatment with an SHP-1 inhibitor. Similarly, TRAF6 levels were measured after treatment with an SHP-1 inhibitor alone. Figure 6c, i shows that the inhibition of SHP-1 increased TRAF6 levels. Similar results were obtained in vitro (Fig. 6b, d, h, j). The overexpression of DJ-1 facilitated the interaction between SHP-1 and TRAF6, which was inhibited after treatment with an SHP-1 inhibitor. However, NLRX1 disassociated from TRAF6 upon DJ-1 overexpression, and SHP-1 inhibition promoted the interaction between NLRX1 and TRAF6 (Fig. 6e, f). Thus, SHP-1 plays a vital role in the disassociation of NLRX1 from TRAF6, which is regulated by DJ-1.

## Discussion

The present study showed that DJ-1 plays an important role in reducing brain injury by inhibiting the levels of inflammatory cytokines in astrocytes. siRNA-induced DJ-1 knockdown increased the death of astrocytes. Reduced DJ-1 expression led to the upregulation of TNF-α, IL-1β, and IL-6. The loss of DJ-1 expression decreased SHP-1 expression and suppressed the interaction between SHP-1 and TRAF6. Conversely, the interaction between NLRX1 and TRAF6 was facilitated. Indeed, DJ-1 induced the disassociation of NLRX1 from TRAF6, which was reversed after the loss of SHP-1. Thus, the anti-inflammatory effects of DJ-1 in astrocytes during cerebral ischemic injury may be due to the induction of NLRX1 and TRAF6 dissociation via SHP-1.

Pathophysiological cascades involving inflammation are triggered by cerebral I/R-induced neuronal death [1, 2]. Cerebral ischemia induces an acute inflammatory response that aggravates tissue damage through elevated production and release of inflammatory cytokines [3, 4]. Acute inflammation elicited by reactive astrocytes after insult is an important response for protecting and repairing the lesion [8]. In addition, the utilization of the secretome of reactive astrocytes was identified as a therapeutic approach for reducing inflammation [9]. Previously, we reported that astrocytes play a critical role in neuroprotection in ischemic injury, which involves DJ-1 [20]. DJ-1 was significantly increased 24 h after reperfusion in reactive astrocytes compared with neurons and microglia in vivo. In addition, in our present study, we showed an important role of astroglial DJ-1 in reducing brain injury.

As a multifunctional protein, DJ-1 possesses many functions, playing roles in processes such as transcriptional regulation [28], antioxidative stress [29], and antiapoptosis [30]. Many previous studies have demonstrated these functions, and new evidence suggests that DJ-1 also has an anti-inflammatory effect. Activated astrocytes produce the proinflammatory cytokines TNF-α, IL-1β, and IL-6 [31], and DJ-1 expression is increased in reactive astrocytes in cerebral I/R injury [20]. In our study, we detected changes in the expression of the cytokines TNF-α, IL-1β, and IL-6 to assess the effect of DJ-1 on inflammation in astrocytes exposed to ischemic injury. The levels of TNF-α, IL-1β, and IL-6 were higher in the DJ-1 knockdown group than in the sham group. High levels of these cytokines can be detrimental to ischemic recovery because they directly induce apoptosis in neuronal cells [32, 33]. In addition, DJ-1 deficiency in other diseases aggravates injury by inducing inflammation [34, 35]. Thus, DJ-1 plays a clear anti-inflammatory role in ischemic injury.

NLRX1, a member of the NLR family of proteins, was recently characterized as a protein that is expressed widely in mitochondria [10, 12]. NLRX1 acts as an anti-inflammatory regulator and inhibits the activation and production of the proinflammatory cytokines TNF-α, IL-1β, and IL-6 [15]. A previous report showed increased proinflammatory cytokine production in Nlrx1−/− mice and aggravated tissue damage during experimental autoimmune encephalomyelitis (EAE) [36]. The anti-inflammatory function of NLRX1 has also been demonstrated in sterile CNS inflammation, such as that in traumatic brain injury [16]. Because DJ-1 is also an anti-inflammatory regulator, we assessed the relationship between DJ-1 and NLRX1 and found NLRX1 expression in mitochondria. Changes in DJ-1 had no effect on NLRX1 levels even though NLRX1 was upregulated under disease conditions. This may be because DJ-1 does not directly regulate NLRX1. NLRX1 associates with TRAF6 in resting cells, but NLRX1 rapidly dissociates from TRAF6 and inhibits NF-κB activation and proinflammatory cytokine release after cell stimulation [17]. Thus, NLRX1 is an anti-inflammatory agent that dissociates from TRAF6 and is critical in regulating inflammation. In our study, DJ-1 induced NLRX1 dissociation from TRAF6. Because DJ-1 had no effect on NLRX1 levels, the molecular mechanisms responsible for the disassociation of NLRX1 from TRAF6 may depend on other molecules or complexes.

SHP-1 is a member of a family of cytosolic protein tyrosine phosphatases [37] that negatively regulate innate and acquired immune responses [38]. The IFN-γ- and LPS-induced activation of NF-κB has been shown to be enhanced in SHP-1-deficient astrocytes [26]. We found that DJ-1 facilitated SHP-1 expression; however, the inhibition of SHP-1 had no effect on the level of DJ-1. Both DJ-1 and SHP-1 play anti-inflammatory roles in ischemic injury. It is possible that DJ-1 exerts anti-inflammatory effects in astrocytes in cerebral I/R injury via SHP-1. A previous study also demonstrated that astroglial DJ-1 exerts anti-inflammatory effects by facilitating the interaction between SHP-1 and STAT1 [23]. The interaction between SHP-1 and TRAF6 is very important for inhibiting inflammation. SHP-1 can also inhibit the production of cytokines induced by Tir by binding to TRAF6 [39]. In addition, SHP-1 directly interacts with TRAF6 in RANKL-stimulated BMMs, and this interaction inhibits downstream signaling molecules such as NF-κB and MAP kinases [27]. We showed an association between SHP-1 and TRAF6 in ischemic injury. DJ-1 promoted the interaction between SHP-1 and TRAF6. Similarly, DJ-1 deficiency significantly suppresses the association of SHP-1 with TRAF6 in RANKL-mediated BMMs [22]. Thus, DJ-1 exerts anti-inflammatory effects in astrocytes in cerebral I/R injury by facilitating the interaction between SHP-1 and TRAF6.

TRAF6, a member of the TNF receptor-associated factor (TRAF) family, is an adaptor that mediates signaling for a host of TNF-R superfamily receptors, including TLRs and NOD-like receptors (i.e., NLRX1), among others [40]. TRAF6 is also known to regulate innate immune responses and proinflammatory cytokines, including NF-κB and IL-6 [41]. We showed that DJ-1 inhibited the expression of TRAF6. In our study, TRAF6 acted as a vital intermediate adaptor involved in a dynamic interaction with SHP-1 or NLRX1. In DJ-1-overexpressing astrocytes, an SHP-1 inhibitor altered the interaction between SHP-1 and TRAF6; therefore, the remaining TRAF6 served as an adaptor scaffold bound to NLRX1. The TRAF6 protein has 4 types of distinct domains: 4 zinc finger (ZF) domains, an N-terminal RING finger domain, a TRAF-C domain, and a coiled-coil domain [42]. A previous study demonstrated that the TRAF6 ZF domain plays a role in SHP-1 recruitment and that the binding of SHP-1 and TRAF6 leads to the deubiquitination of TRAF6 and inhibits NF-κB activity in RANKL-activated BMMs [39]. TRAF6 is a ubiquitin (Ub) ligase that activates signaling via the attachment of K63-linked Ub chains, and a class of deubiquitinating enzymes that disassemble these chains to negatively modulate signaling has been identified [43]. In our study, the binding between SHP-1 and TRAF6 may have led to the deubiquitination of TRAF6 and the subsequent disassembly of the TRAF6-SHP-1 complex. These results suggest that the dynamic interaction between TRAF6 and NLRX1 or SHP-1 may be more complicated than previously believed. Thus, further studies to determine whether the binding between SHP-1 and TRAF6 leads to the deubiquitination of TRAF6 and whether the deubiquitination of TRAF6 affects the binding between TRAF6 and NLRX1 are needed.

## Conclusion

**Fig. 7:**
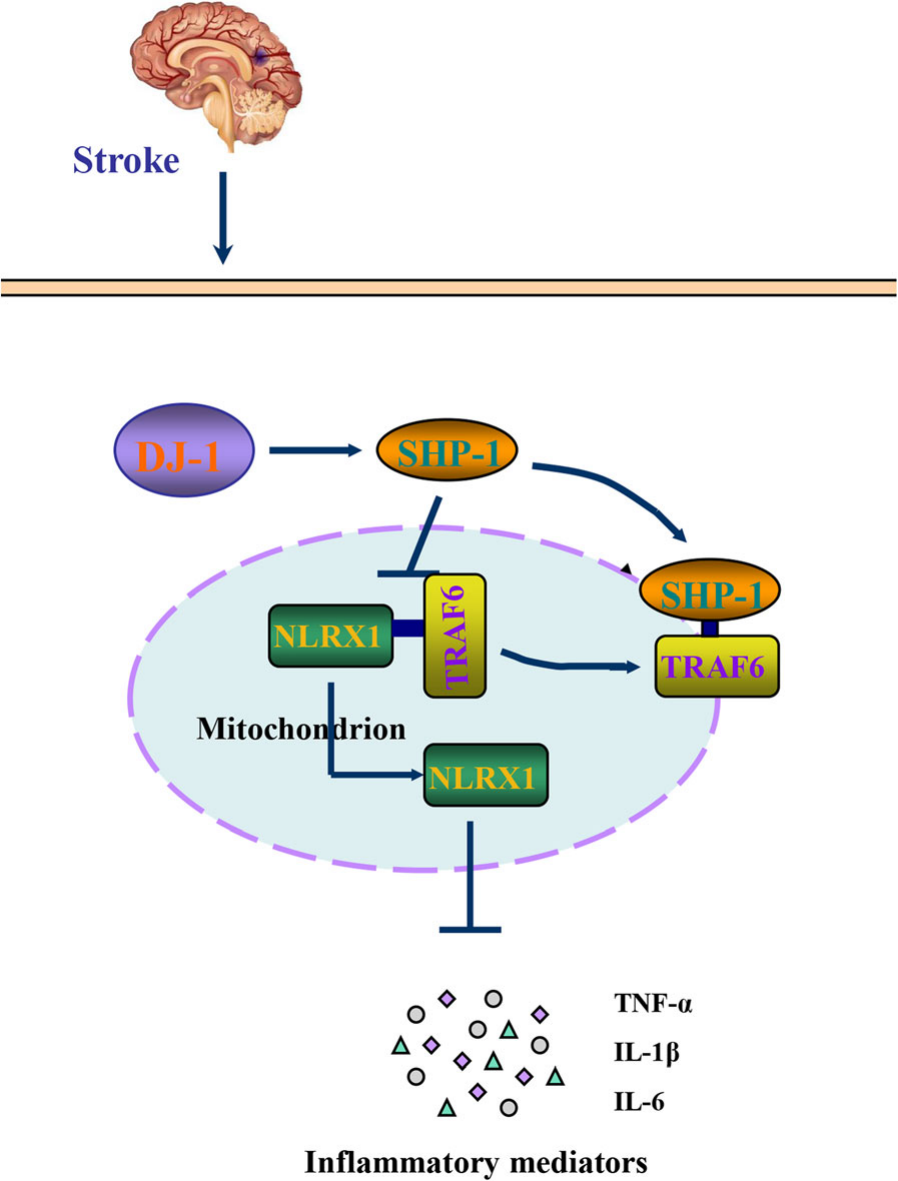
Mechanisms by which DJ-1 regulates inflammation. DJ-1 exerts an anti-inflammatory effect by decreasing the expression of cytokines IL-1β, IL-6, and TNF-α in cerebral I/R injury. DJ-1 facilitates the dissociation of NLRX1 from TRAF6. DJ-1 upregulates SHP-1 expression and facilitates the interaction between NLRX1 and TRAF6. DJ-1 induces the dissociation of NLRX1 from TRAF6 via SHP-1

The findings presented here identify a previously unrecognized role for DJ-1 in exerting anti-inflammatory effects in astrocytes during cerebral I/R injury by regulating the interaction between SHP-1 and TRAF6, thereby mediating the disassociation of NLRX1 from TRAF6. The mechanism is shown in Fig. 7. Thus, DJ-1 may be an efficacious therapeutic target for treating I/R injury.

## Data Availability

The datasets supporting the conclusions of this article are included within the article.

## Abbreviations

I/R: Cerebral ischemia/reperfusion
SHP-1: Src homology region 2 domain-containing phosphatase-1
TNF-α: Tumor necrosis factor-α
TPI-1: SHP-1 inhibitor
TRAF6: TNF receptor-associated factor 6
